# South West Urologists Club, Abstracts of Meeting at Taunton

**Published:** 1987-05

**Authors:** 


### South West Urologist's Club

*Meeting at Taunton April 1986*
*Abstracts of papers*

### IMPAIRED SPERMATOGENESIS IN TESTES AT RISK OF TORSION

**J. B. Anderson, W. E. G. Thomas, R. C. N. Williamson, P. J. B. Smith**
**Departments of Surgery & Urology, Bristol Royal Infirmary**

Although immunological sympathetic orchidopathia has been suggested as a cause for the oligospermia observed after spermatic cord torsion, there may be an underlying testicular abnormality causing decreased spermatogenesis in these patients.

To investigate this possibility 20 post-pubertal men with acute torsion were studied prospectively to correlate the histological appearances of immediate contralateral testicular biopsy with testicular function 3 months later.

Thirteen of 20 patients had evidence of partial maturation arrest in spermatogenesis which was either mild (n=6), moderate (4) or severe (3). All 7 patients with normal histology had a sperm concentration greater than $25 \times 10^6$/ml after 3 months, but 10 of 13 with maturation arrest were oligospermic ($<20 \times 10^6$/ml). The Johnsen score (1) in patients with a normal biopsy (median 9.01; s.q. range 8.96–9.21) was higher than in those with abnormal histology (median 8.28; s.q. range 7.98–8.45; $p<0.001$) and correlated with the log of the sperm concentration 3 months later ($r=0.79$; $p<0.001$). There was no anti-sperm or anti-testis antibody formation following torsion, and testicular endocrine function remained normal.

This study demonstrates that most patients presenting with spermatic cord torsion have an underlying abnormality of the testis which correlates well with its poor postoperative exocrine function. No evidence of immunological sympathetic orchidopathia was demonstrated but the contralateral testicular damage seen in animal experiments and the association between the duration torsion and the degree of oligospermia (2) remains unexplained.

### REFERENCES

1. Johnsen, S. G. Testicular biopsy score count—a method for registration of spermatogenesis in human testes. Hormones 1970; 1: 2–25.
2. Thomas, W. E. G., Cooper, M. J., Crane, G. A., Lee, G., Williamson, R. C. N. Testicular exocrine malfunction after torsion. Lancet 1984; ii: 1357–60.

### THE PATHOPHYSIOLOGY OF ACUTE RETENTION

**J. A. Massy, FRCS**
**Clinical Investigation Unit, Ham Green Hospital, Pill, Bristol**

Sixty men, mean age 71 years, presenting in retention due to benign prostatic hypertrophy with no obvious precipitating event were studied. A survey of all admissions showed that the peak presentation was in the seventh decade and compared to 'cold prostatic' patients, the history was short and obstructive rather than irritative.

Urodynamic studies were performed on admission, after decompression by suprapubic catheter and over 72 hours. Retention cystograms showed closure of the bladder neck area even onstraining. The urethral profile was of normal pattern in 60% but showed significant elevation of the plateau level and shortening of prostatic length ($p<0.05$) compared to decompression values. Some authorities have proposed sympathetic overactivity as the cause of retention. However, the mid-prostatic peak produced in this situation is only present in 40%, all of whom had a greater retained volume ($p<0.01$). These findings suggest that simple compression from the distended bladder produces the recorded prostatic changes. There was no evolution of urethral profile parameters following decompression and histological studies showed no evidence of acute changes in the prostate. On the other hand, the detrusor was decompensated and gradually recovered normal function so that 80% of patients were able to produce some degree of voiding at 4 days. In 20% surgery was not indicated. No factors predicted the recovery of micturition.

It is postulated that in a population with 'under-active' detrusor function, the response to progressive obstruction is decompensation resulting in retention.

### SECOND MALIGNANCIES—A METHOD OF ESTIMATING MUTAGENIC POTENTIAL OF ANTI-CANCER DRUGS

**M. J. Phillips, Consultant Haematologist, Somerset**

Prolonged survival of patients with haematological malignancies treated with chemotherapeutic agents has become increasingly likely in the past twenty years. However, there is evidence that successfully treated patients seem to have increased risk of developing subsequent malignancies, eg, Hodgkin's disease and increased frequency of later developing acute myeloid leukaemia. One explanation is that the drugs used successfully to treat one malignancy produce genetic changes which result in secondary or tertiary cancers. Certainly there is an accepted link between mutagenesis and carcinogenesis. Most studies have concentrated on demonstrating cytogenic injury but here it is difficult to discriminate between cause and effort. Many drugs have been screened for mutagenic effects by measuring mutations in bacteria but there are inherent defects in such studies, not the least being the high spontaneous mutation rate in such test systems.

This paper reports the results of some of the studies carried out by the Taunton and Exeter research team as part of a larger study of mutagenicity. A system was devised whereby cultured mammalian cells, Chinese Hamster V79 cells, were co-cultured with rat hepatocytes. This allowed hepatic metabolism of drugs added to the system. Spontaneous mutation rates in the V79 cells are low and this particular cell line is easily scored for mutation by the development of cells exhibiting resistance to 6-thioguanine. The system also lends itself to toxicity studies which are always carried out in concert with measurement of mutagenic effect.

Six drugs commonly used in treating haematological malignancies have been studied and reported on. Cyclophosphamide and Adriamycin were strongly mutagenic and 6-mercaptupurine weakly so. Cytosine Arabinoside showed a slight mutagenic effect but both Methotrexate and Vincristine were non-mutagenic. All of these drugs were toxic to the mammalian cells but the response was determined by the presence or absence of co-cultured hepatocytes. The results indicate that this is a viable and sensitive test of the mutogenic potential of anti-cancer drugs. The co-culture of mammalian cells with hepatocytes plainly allows both the necessary metabolism of certain drugs to produce their cytotoxic metabolites, eg, Cyclophosphamide or the detoxification of others, eg, Adriamycin. The toxicity studies may provide valuable indications of optimal dose selection of the drugs tested.

### TEFLON INJECTION IN FEMALE STRESS INCONTINENCE

**S. Vesey, A. Rivett, P. O'Boyle**
**Department of Urology, Musgrove Park Hospital Taunton**

Thirty six females with Genuine Stress Incontinence were treated by perurethral bladder neck Teflon (Polytef) injection. The injections were given using a purpose built Teflon injecting endoscope at 3, 6 and 9 o'clock, submucosally, immediately below the bladder neck. The average volume of Teflon injected was 7 ml. Post operative catheters were not used. The procedure was repeated in 8 cases.

All bladders were stable on pre-operative urodynamic assessment. Eighteen patients had both pre- and post-operative Urethral Pressure Profile studies (UPPs). Post-operative UPP studies were carried out at 1, 3 and 6 months. Eighteen of the patients had previously failed to respond to an open anti-incontinence procedure. The mean patient age was 55 years (range 30–80 years). The average follow up was 9 months (range 2–36 months).

Twenty patients were cured i.e. completely dry on follow up, 4 were improved and 12 failed to respond. The procedure was deemed to have been successful if the patient was cured or improved by the injection. The overall success rate was 67%. Of the eighteen that had previous anti-incontinence surgery 14 (78%) had a successful outcome.

Urethral pressure profilometery showed that bladder neck Polytef injection increased the functional profile length of the urethra by 10% over it's pre-operative measurement. There was no demonstrable change in the maximum urethral closure pressure following injection.

The procedure was safe, simple, readily repeated and gave best results in those who had previously failed to respond to open anti-incontinence surgery.

### THE USE OF PROSTATIC-SPECIFIC ANTIGEN IN PROSTATIC CARCINOMA

**M. Ferro, I. Barnes*, J. B. M. Roberts, P. J. B. Smith**
**Departments of Urology and Biochemistry*, Bristol Royal Infirmary**

A study has been performed on 130 men comparing the level of serum Prostate-Specific Antigen (PSA) in control males, patients with benign prostatic hyperplasia (BPH) and patients with prostatic carcinoma. The results have shown firstly that all 30 normal male controls below 40 years had values less than 10 ng/ml. Of the 40 patients with BPH all over 40 years 13 out of 40 (32.5%) had raised levels above 10 ng/ml. In the 60 patients with prostatic carcinoma, again all over 40, 24 had localised disease (M0) and 36 had metastatic spread (M1), as judged by isotope bone scan. In those patients with M0 disease, 16 out of 24 (66.6%) had raised PSA levels compared with 34 out of 36 (94.5%) of patients with M1 disease. The corresponding figures for raised prostatic acid phosphatase (PAP) values were 4% in the M0 group, and 52.7% in the M1 group.

PSA levels did not reflect the histologic grade nor the local stage of the tumour and was of no value in estimating tumour burden.

However, in the management of prostatic cancer PSA was found to be a valuable index due to this sensitivity. Stable disease not requiring hormonal manipulation was reflected by unchanging levels of PSA whereas progressive disease requiring hormonal therapy was reflected by an alteration in the PSA levels corresponding to the patients' response. The same group of progressive disease patients showed only a 50% rise in serum PAP levels confirming the greater sensitivity of PSA as a measure of prostate cancer.

PSA measurements should be included in any further trials on prostatic carcinoma and should replace PAP as the standard marker for evaluating response to therapy.

### B5 TUMOUR MARKER IN TCC BLADDER

**K. Malpani, A. Hinchliffe**

A study of B5 antigen has been applied to new and old patients undergoing cystoscopy for TCC bladder and compared with urinary cytology. The original study of B5 and TCC bladder was carried out in Cambridge and samples of blood from our patients were also tested in Cambridge.

B5 antigen detected by haemaflutination occurs naturally but weakly in most controlled subjects (80%) but positivity usually increases markedly in tumour bearing patients regardless of tumour type. More than 30% of erythrocytes agglutinating in this study is expressed as positive.

The original Cambridge study showed a high degree of B5 positivity in TCC patients and in some this reduced after successful tumour treatment.

The initial results from three patient groups in our study showed:—

1. A similar pattern of B5 positivity to those in the Cambridge study though shifted towards the negative (? erythrocyte deterioration in transit).
2. Urine cytology was rather better at predicting tumour presence of absence.
3. When both indicators were in accord the accuracy of prediction was enhanced.

### Weston study

| Cystoscopy findings | | % Cytology malignant | % B5 positive |
|---|---|---|---|
| New tumours | 32 | 82 | 66 |
| Recurrences | 68 | 66 | 54 |
| No recurrences | 79 | 37 | 45 |

### Cambridge study

| Cytoscopy | | % B5 positive |
|---|---|---|
| New tumours | 20 | 90 |
| Recurrences | 54 | 76 |
| No recurrences | 24 | 45 |

### REFERENCES

Metcalfe, S. M., Jamieson, N. V. (1984) Annals of the Royal College of Surgeons of England. 1984; **66**: 401.

### ENDOSCOPIC TEFLON INJECTION FOR VESICOURETERIC REFLUX

**M. J. Stower, J. C. Gingell**
**Department of Urology, Southmead Hospital**

The management of vesicoureteric reflux has always been a controversial subject, especially the indications for surgery. O'Donnell and Puri (1) described an endoscopic method of injecting Teflon paste behind the intravesical ureter, curing reflux in 14 out of 18 ureters. Since 1985 we have used this method in selected patients.

Nine female patients (median age 10 years: range 7–48 years) with vesicoureteric reflux have been treated so far. The patients were admitted as a day case and under general anaesthesia, 0.5 ml–1.0 ml of polytef paste (Ethicon) was injected 2–3 mm below the ureteric orifice at a depth of 0.5 cm–1.0 cm using the specially designed Wolf Teflon injector set. A urethral catheter was left in situ for a post operative micturating cystogram (MCUG) and following this an IVU was performed. The post operative MCUG showed that reflux had been cured in 8 out of 13 ureters, improved from grade III to grade I in a further 3, unchanged in the other 2. None of the IVU's showed any evidence of ueteric obstruction. Eight of the patients are no longer taking prophylactic antibiotics.

Endoscopic Teflon injection has proved to be an efficient and cost effective way of managing vesicoureteric reflux with no immediate post operative complications and with the potential of repeating the procedure if necessary.

### REFERENCE

(1) O'Donnell, B., Puri, P. (1984) Treatment of vesicoureteric reflux by endoscopic injection of Teflon. Brit. med. J. **289** 7–9.

### OUTPATIENT OPS.

At the outpatient ops you just cut off their tops,
You are destiny shaping their ends,
Taking each little man you snip off what you can,
Then you send him outside to his friends. . . .
Though it seems a bit thick, they are bound to be sick,
And the bandage drops off with the very first cough,
And the stitches slough out when they're crawling about.,
Which is not very hard to explain.

In a very short time when you're reaching your prime,
And you're doing the dressings one day,
You will get a rude blow when you look – well you know,
And you see – well I'd rather not say!
And the parents complain that the child is in pain,
For it's covered with dirt and stuck fast to his shirt;
Its quite obvious why he is starting to cry,
And it's not very hard to explain.

GEORGE ALEC DIBB

Note – This was written when George Dibb was a student at the General Infirmary at Leeds in the 1920s. It was published in 'Aegophany', a rollicking anthology of student verse, much of it written to be sung with well known tunes at the annual pantomime. At this time circumcision was almost a ritual amongst Christians as well as amongst Jews, but it was done not in the newborn by the Rabbi but at about 6 months by medical students 'under instruction', sometimes with results 'which were not very hard to explain'. Ed.

